# Patient safety culture among paramedic university students in Saudi Arabia

**DOI:** 10.1371/journal.pone.0344539

**Published:** 2026-03-19

**Authors:** Hussin Albargi

**Affiliations:** Programme of Emergency Medical Service, College of Nursing and Health Sciences, Jazan University, Jazan, Saudi Arabia; King Abdulaziz University Faculty of Medicine, SAUDI ARABIA

## Abstract

**Background:**

A strong and well-established patient safety culture is a fundamental component of healthcare systems and is especially vital in Emergency Medical Services (EMS). Understanding the attitudes of paramedic students toward patient safety offers valuable insights into their preparedness and highlights potential gaps in educational curricula requiring targeted enhancement.

**Objective:**

This study aimed to evaluate the patient safety attitudes among paramedic university students in Saudi Arabia and explore differences based on demographic factors, including gender, academic year, and academic performance.

**Methods:**

A cross-sectional survey was conducted with 494 paramedic students using the Safety Attitudes Questionnaire (SAQ), covering six domains: Teamwork Climate, Safety Climate, Job Satisfaction, Stress Recognition, Perception of Management, and Working Conditions. Statistical analyses included descriptive statistics, chi-square tests, ANOVA, and Bonferroni post-hoc comparisons.

**Results:**

A total of 494 paramedic students participated. Overall, 25.1% achieved a positive safety attitude (SAQ ≥ 75). Among domains, Job Satisfaction scored highest (76.2 ± 15.2), while Stress Recognition scored lowest (51.2 ± 28.1). Significant differences were observed across academic years, with interns demonstrating higher domain scores than fourth-year students in teamwork climate, safety climate, stress recognition, job satisfaction, and perception of management (p < 0.01).

**Conclusion:**

Paramedic students demonstrated low overall safety attitudes, with particularly low scores in stress recognition and reduced perceptions among fourth-year students. Strengthening stress management, teamwork, and supervisory support during training, alongside organisational efforts to enhance the clinical learning environment, may help improve the patient safety culture in paramedic education.

## Introduction

Patient safety is a core dimension of healthcare quality and remains a global priority given the scale of preventable harm. An estimated one in ten patients experiences harm during care, and unsafe care is associated with more than three million deaths annually; in low- and middle-income countries, up to four in 100 people may die from unsafe care [1]. Addressing this burden requires a well-developed patient safety culture, defined as the shared values, attitudes, and practices that prioritise safe and effective care delivery.

Emergency medical services (EMS) personnel deliver care in dynamic, unpredictable, and resource-limited conditions that require rapid decisions, often with incomplete information. The prehospital environment is recognised as a high-risk domain for error yet remains comparatively underexamined. A systematic review identified 88 studies and grouped threats into seven recurrent areas: adverse events and medication errors, clinical judgement, communication problems (including information loss at handover), ground and air transport safety, interfacility transfer, and airway management [2]. These features heighten the likelihood of failures in assessment, dosing, equipment use, and transitions of care and make a strong safety culture within EMS imperative.

Patient safety attitudes represent a measurable dimension of safety climate, reflecting perceptions of teamwork, communication, leadership, and organisational support for safe practice. In prehospital settings, where care is delivered under time pressure and variable supervision, these perceptions can influence behaviours such as speaking up, error reporting, and adherence to protocols. Examining safety attitudes during training, therefore, provides insight into how future paramedics interpret and engage with safety norms in high-risk clinical environments.

The high-risk nature of EMS practice raises important questions about how patient safety culture, as a collective and institutionally embedded phenomenon, is shaped during training. While much of the literature has examined safety culture among practicing healthcare professionals, comparatively little is known about students, particularly those preparing for careers in paramedicine. The academic and clinical preparation of paramedic students is a formative period that influences their professional identity and future practice behaviours [3]. Limited exposure to patient safety principles during this stage may contribute to inconsistent safety attitudes and reduced preparedness for clinical responsibilities [4]. In addition, paramedic students face considerable psychological and educational pressures, including exposure to critical incidents, heavy academic workloads, and the transition into high-stakes clinical placements [4,5]. These stressors can impair cognitive performance, emotional regulation, and adherence to safety protocols. Understanding how training experiences interact with safety attitudes is therefore essential for designing educational strategies that strengthen the foundations of patient safety in prehospital care.

Despite the growing role of EMS in Saudi Arabia, little research has examined patient safety culture among paramedic students [6]. Most existing work has focused on medical and dental students, leaving those preparing for prehospital care largely overlooked. This study employs the Safety Attitudes Questionnaire to assess patient safety attitudes among paramedic university students in Saudi Arabia and to explore differences by gender, academic year, and academic performance [7]. The findings aim to guide curriculum development and strengthen the preparation of a workforce capable of upholding patient safety in high-risk clinical environments.

## Methods

### Study design, setting, and population

This cross-sectional study was conducted between January 20 and March 20, 2025, among undergraduate paramedic students in Saudi Arabia. Data were collected using an online self-administered survey distributed electronically to paramedic students across multiple universities through official programme and institutional channels. Institution-level identifiers and the number of respondents per university were not collected to preserve institutional anonymity and encourage participation. Eligible participants included all students enrolled in paramedicine programs during the study period, and a total of 494 students completed the questionnaire. For analysis, academic year categories were adjusted due to small group sizes, with second- and third-year students combined into a single group. Paramedic education programmes in Saudi Arabia follow an undergraduate structure with progressive clinical exposure. Early academic years focus primarily on classroom-based learning with limited clinical practice, while later years involve increased clinical placements in both prehospital and hospital settings. The final year typically consists of a full-time internship with sustained clinical immersion. These differences are relevant when interpreting variations in patient safety attitudes across academic years.

### Data collection tool

Patient safety attitudes were measured using the Safety Attitudes Questionnaire (SAQ), a psychometrically robust and internationally validated tool widely utilized in healthcare research [7,8]. In this study, the SAQ was organized into six domains: teamwork climate (6 items), safety climate (7 items), job satisfaction (5 items), stress recognition (4 items), perception of management (5 items), and working conditions (4 items). Each item was rated on a five-point Likert scale (1 = strongly disagree to 5 = strongly agree) and subsequently transformed into a 0–100 scale, with higher scores reflecting more positive safety attitudes. A mean score of ≥75 was adopted, in line with established conventions, as the threshold for a “positive” patient safety attitude, providing a benchmark to identify both areas of strength and domains requiring improvement in safety culture [7]. Prior to the main data collection, the questionnaire was pilot-tested with a small group of paramedic students to assess clarity and feasibility. No substantive modifications were required, and pilot data were not included in the final analysis. In the Saudi paramedic curriculum, students undertake a substantial proportion of their clinical training in hospital-based settings in addition to prehospital placements.

### Data analysis

Descriptive statistics (frequencies, percentages, means, and standard deviations) were used to summarize participant characteristics and SAQ domain scores. Associations between demographic factors and positive safety attitudes (SAQ ≥ 75) were examined using chi-square or Fisher’s exact tests as appropriate. Differences in mean SAQ domain scores across academic years were analysed using one-way ANOVA with Bonferroni post-hoc comparisons. All statistical tests were two-sided, and statistical significance was set at p < 0.05. Data were analysed using stata version 16 (stataCorp, College Station, TX, USA).

### Ethical considerations

The study protocol was reviewed and approved by the Institutional Review Board at the University of Jazan, Saudi Arabia (Reference No.: REC-46/07/1359). Participation was voluntary. Informed consent was obtained electronically at the start of the online survey, where students were presented with a participant information statement and asked to indicate their agreement before accessing the questionnaire. Consent was inferred from agreement and survey completion. No identifying information was collected, and all responses were anonymised.

## Result

### Participant characteristics

A total of 494 paramedic students completed the survey. The majority were male (84.0%) and aged 22–23 years (60.3%). Most participants were in their fourth year (49.2%), followed by the third year (26.7%) and internship year (24.1%). Academic performance was generally high, with 75.5% of students reporting a GPA of 4.0 or higher (Table 1).

**Table 1:** Demographic and academic characteristics of paramedic students.

Characteristic	Category	n	%
**Gender**	Male	415	84.0%
	Female	79	16.0%
**Age Group**	≤21	172	34.8%
	22–23	298	60.3%
	≥24	24	4.9%
**Academic Year**	Second/Third year	132	26.7%
	Fourth year	243	49.2%
	Internship	119	24.1%
**GPA Category**	<3.50	28	5.7%
	3.50–3.99	93	18.8%
	4.00–4.49	183	37.0%
	4.50–5.00	190	38.5%

### Safety attitudes questionnaire scores

**Fig 1:**
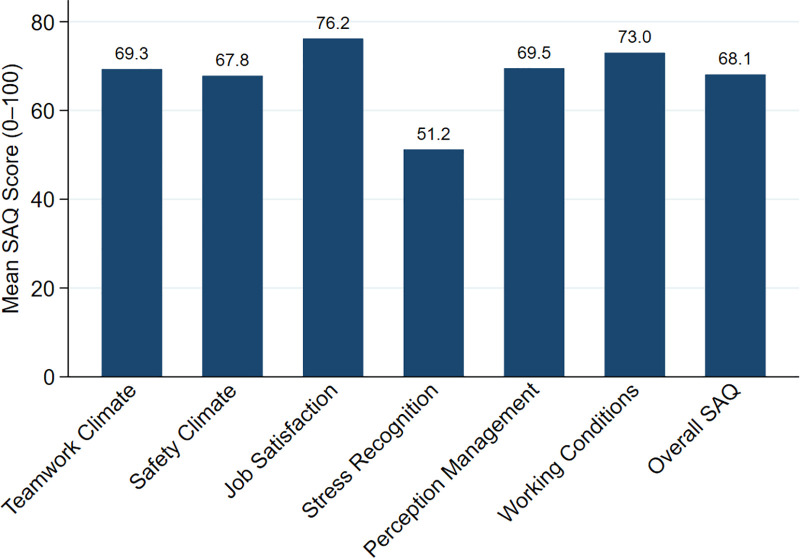
Mean safety attitudes questionnaire (SAQ) domain scores among paramedic students (n = 494).

The mean Safety Attitudes Questionnaire (SAQ) scores across domains are presented in **Fig 1**. Job satisfaction recorded the highest score (76.2 ± 15.2), while stress recognition was the lowest (51.2 ± 28.1). Teamwork climate (69.3 ± 12.3), safety climate (67.8 ± 12.6), perception of management (69.5 ± 12.6), and working conditions (73.0 ± 14.5) showed intermediate values. The overall SAQ mean score was 68.1 ± 11.7. A total of 25.1% of students achieved a positive safety attitude (SAQ ≥ 75).

### Associations with demographic characteristics

Positive safety attitudes were significantly associated with gender, age, and academic year (**Table 2**). Male students reported higher rates of positive attitudes compared with females (28.2% vs. 8.9%, p < 0.001). Students aged ≤21 years showed the highest proportion of positive attitudes (36.6%) compared with 22–23 years (17.8%) (p < 0.001). Across academic levels, interns reported the highest proportion of positive attitudes (42.0%) relative to third-year (27.3%) and fourth-year students (15.6%) (p < 0.001). No significant differences were observed across GPA categories (p = 0.856).

**Table 2:** Association between participant characteristics and positive safety attitude (SAQ ≥ 75).

Characteristic	Category	N (%) Positive SAQ	N (%) Negative SAQ	p-value
**Gender**	Male	117 (28.2%)	298 (71.8%)	**<0.001**
	Female	7 (8.9%)	72 (91.1%)	
**Age Group**	≤21	63 (36.6%)	109 (63.4%)	**<0.001**
	22–23	53 (17.8%)	245 (82.2%)	
	≥24	8 (33.3%)	16 (66.7%)	
**Academic Year**	Third	36 (27.3%)	96 (72.7)	**<0.001**
	Fourth	38 (15.6%)	205 (84.4%)	
	Internship	50 (42.0%)	69 (58.0%)	
**GPA Category**	<3.50	8 (28.6%)	20 (71.4%)	0.856
	3.50–3.99	26 (28.0%)	67 (72.0%)	
	4.00–4.49	44 (24.0%)	139 (76.0%)	
	4.50–5.00	46 (24.2%)	144 (75.8%)	

### Pairwise comparisons across academic years

Post-hoc Bonferroni analyses revealed several significant differences in SAQ domain scores across academic levels (**Table 3**). Teamwork climate and safety climate scores were significantly higher among third-year students compared with fourth-year students (p < 0.01), and interns scored higher than fourth-year students in both domains (p < 0.001). Job satisfaction was significantly higher among interns compared with fourth-year students (p < 0.01). In stress recognition, both third-year students (p < 0.05) and interns (p < 0.01) reported higher scores than fourth-year students. For perception of management, interns scored significantly higher than both third-year (p < 0.01) and fourth-year students (p < 0.001). No significant differences were observed across academic years for working conditions.

**Table 3:** SAQ domain scores by academic year and significant differences.

Domain	Third Year	Fourth Year	Internship	p-value	Significant Differences^a^
**Teamwork Climate**	70.4 ± 12.1	66.5 ± 12.9	73.7 ± 10.2	<0.001	T > F**, I > F***^b^
**Safety Climate**	69.3 ± 12.7	64.9 ± 12.4	72.2 ± 10.1	<0.001	T > F**, I > F***
**Job Satisfaction**	77.1 ± 13.4	74.0 ± 16.9	79.6 ± 12.7	0.003	I > F**
**Stress Recognition**	54.5 ± 27.6	46.6 ± 26.4	57.0 ± 30.5	0.001	T > F*, I > F**
**Perception of Management**	69.6 ± 13.1	67.1 ± 12.1	74.3 ± 11.5	<0.001	I > T**, I > F***
**Working Conditions**	73.9 ± 14.6	72.1 ± 15.1	73.9 ± 13.3	0.396	—

^a^*p < 0.05, **p < 0.01, ***p < 0.001**,** ^b^T = Third Year, F = Fourth Year, I = Internship.

## Discussion

This study evaluated patient safety culture among paramedic university students in Saudi Arabia using the Safety Attitudes Questionnaire (SAQ). The findings demonstrate relatively low overall safety attitudes, with only one quarter of students achieving a positive SAQ score. Among the SAQ domains, job satisfaction scored highest, while stress recognition was lowest, highlighting variability across safety-related perceptions. Importantly, this is the first study to assess patient safety culture specifically among paramedic students in Saudi Arabia, addressing a critical gap in the literature. These findings should be interpreted as reflections of students’ perceptions of their educational and clinical environments rather than as individual traits, and lower scores among certain groups may indicate heightened critical awareness of safety challenges rather than poorer individual commitment to patient safety.

Compared with other student populations in Saudi Arabia, paramedic students in this study reported less favourable safety attitudes. Dental students in Dammam also reported higher safety culture means, with teamwork and safety climate often exceeding 70 points [9]. In contrast, only 25.1% of paramedic students in the present study achieved a positive SAQ score, with stress recognition particularly low at 51.2. These differences may reflect several factors. Paramedic education in Saudi Arabia is relatively new and may not yet fully embed patient safety within its curricula. The prehospital training context, characterized by unpredictability, limited supervision, and time pressure, may further hinder the development of positive safety attitudes. Stressors such as workload, exposure to emergencies, and clinical transition have been shown to affect cognitive performance and safety practices [4]. International studies echo these contrasts: EMS professionals in Finland reported domain means ranging from 63.1 to 79.2, and U.S. EMS personnel averaged ~72 across domains, both higher than those of the present cohort [10,11]. Together, these comparisons suggest that weaker safety perceptions among paramedic students may stem from both educational gaps and the inherent challenges of prehospital care.

In this study, male students demonstrated significantly higher positive safety attitudes compared with female students (28.2% vs. 8.9%). This difference may partly be explained by the small proportion of female participants, who represented only 16% of the sample, a pattern consistent with the broader gender imbalance in paramedic education and practice in Saudi Arabia [6,12,13]. Although opportunities for women in paramedicine have recently expanded, cultural perceptions and systemic barriers continue to restrict their integration, and public surveys reveal mixed acceptance of female paramedics in the Kingdom [13–15]. Similar gender-based patterns have been reported in Saudi medical education, where male students expressed more positive attitudes in domains such as teamwork and safety [16]. By contrast, research in pharmacy education found female students to report stronger attitudes in areas such as error reporting and teamwork, while Saudi nursing students showed differences in teamwork competence favouring males, with overall competence improving during internship [17,18]. Taken together, these findings suggest that gender disparities in safety attitudes are multifactorial: they stem not only from unequal representation but also from sociocultural dynamics that continue to shape women’s experiences in health professions.

A notable feature of our results is the variation in SAQ domain scores across academic years, with fourth-year students consistently scoring lower than both third-year and internship groups. This “dip” in safety attitudes may reflect the transitional challenges of the fourth year, when students face heavier academic loads and demanding clinical expectations while preparing for graduation. The fourth year is particularly high-stakes, as students balance the culmination of theoretical coursework with the pressures of clinical placements, often without the structured immersion and mentorship afforded during the internship year. Such circumstances may foster stress, uncertainty, and early signs of burnout, which are known to negatively influence performance and safety-related attitudes [19,20]. Similar declines have been observed in Saudi dental and nursing education, where mid-training students reported lower confidence and weaker safety perceptions compared with interns or final-year students [9,21]. By contrast, the higher scores among interns likely reflect increased professional identity formation, greater experiential learning, and a sense of belonging to clinical teams, all of which reinforce positive safety perceptions [3]. Interns reported more positive perceptions of management, which may reflect differences in supervisory structures during the internship year. Unlike in earlier academic years, interns are primarily supervised by clinical supervisors and preceptors in healthcare and emergency medical services settings, where leadership roles, expectations, and feedback mechanisms are more clearly defined. This distinction in the management context may partially explain higher scores in this domain.

The findings of this study indicate the need for coordinated strategies to strengthen patient safety culture within paramedic training contexts in Saudi Arabia. Given the low overall proportion of positive safety attitudes and the variation observed across academic years and domains, interventions should extend beyond curricular content alone. Structured patient safety teaching and simulation-based reinforcement may support the development of safety awareness, particularly in domains such as stress recognition and safety climate. However, parallel attention to the quality of clinical learning environments, including supervision practices, feedback mechanisms, and team integration during placements, is likely necessary to support more consistent safety perceptions. Addressing both educational and organisational dimensions may help foster a more resilient safety culture among future paramedics.

### Strengths and limitations

This study is the first to assess patient safety culture among paramedic university students in Saudi Arabia, addressing an important gap in the literature on prehospital emergency care education. The use of the validated Safety Attitudes Questionnaire (SAQ) enhances comparability with national and international studies.

However, several limitations should be acknowledged. The cross-sectional design precludes inference of temporal or causal relationships between student characteristics and safety attitudes. The reliance on self-reported responses introduces the possibility of social desirability bias and other response biases. In addition, the exclusive use of a single survey instrument without methodological triangulation (e.g., qualitative data or observational measures) may limit the depth of interpretation. Although students were recruited from multiple universities, institution-level identifiers were not collected; therefore, potential variability across training environments could not be examined, potentially affecting generalizability. Finally, the relatively small proportion of female students limits the robustness of gender-based comparisons.

## Conclusion

This study is the first to examine patient safety culture among paramedic students in Saudi Arabia, providing novel insights into a population that has received little research attention. The results indicate that while some domains such as job satisfaction were rated positively, overall safety attitudes remain low, with pronounced differences across academic years. These findings suggest that the development of safety culture is not uniform during training and may be shaped by the balance between academic pressures and clinical exposure.

To advance safety competence among future emergency care providers, paramedic education programs should prioritize longitudinal integration of patient safety principles, beginning early in training and reinforced progressively through applied learning. Simulation-based practice, structured mentorship, and consistent opportunities for reflective engagement with safety concepts may be especially valuable. Investing in these approaches can help build a workforce prepared to deliver safe, reliable care in the complex and unpredictable prehospital environment.

## Supporting information

S1 Table Safety attitudes questionnaire. (DOCX)
