# Supplementary material for: Patient safety culture among paramedic university students in Saudi Arabia
Source: PLoS One. 2026 Mar 19;21(3):e0344539. doi: 10.1371/journal.pone.0344539 (PMC13001958; doi:10.1371/journal.pone.0344539)
Supplement: S1 Table — (DOCX) [file pone.0344539.s001.docx]

**Safety Attitudes Questionnaire (SAQ) Domains, Items, Mean Scores, and Percentages of Positive Responses**

| **Domain / Item** | **Mean** | **SD** | **% Positive Responses** |
| --- | --- | --- | --- |
| **Job Satisfaction** |  |  |  |
| I like my job |  |  |  |
| Working here is like being part of a large family |  |  |  |
| This is a good place to work |  |  |  |
| I am proud to work in this clinical area |  |  |  |
| Morale in this clinical area is high |  |  |  |
| **Safety Climate** |  |  |  |
| I would feel safe being treated here as a patient |  |  |  |
| Medical errors are handled appropriately in this clinical area |  |  |  |
| I know the proper channels to direct questions regarding patient safety in this clinical area |  |  |  |
| I receive appropriate feedback about my performance |  |  |  |
| In this clinical area, it is difficult to discuss errors |  |  |  |
| I am encouraged by my colleagues to report any patient safety concerns I may have |  |  |  |
| The culture in this clinical area makes it easy to learn from the errors of others |  |  |  |
| **Teamwork Climate** |  |  |  |
| Student’ input is well received in this clinical area |  |  |  |
| In this clinical area, it is difficult to speak up if I perceive a problem with patient care |  |  |  |
| Disagreements in this clinical area are resolved appropriately (i.e., not who is right, but what is best for the patient) |  |  |  |
| I have the support I need from other personnel to care for patients |  |  |  |
| It is easy for personnel here to ask questions when there is something they do not understand |  |  |  |
| The supervisors and students here work together as a well-coordinated team. |  |  |  |
| **Working Conditions** |  |  |  |
| Problem personnel are dealt with constructively by our clinical units. |  |  |  |
| This clinical area does a good job of training new personnel. (eg students or staff). |  |  |  |
| All the necessary information for diagnostic and therapeutic decisions is routinely available to me |  |  |  |
| Trainees in my discipline are adequately supervised |  |  |  |
| **Perception of Management** |  |  |  |
| Management supports my daily efforts |  |  |  |
| Clinical management doesn't knowingly compromise patient safety. |  |  |  |
| I get adequate, timely info about events that might affect my work. |  |  |  |
| The levels of students in my clinical area are sufficient to handle the number of patients. |  |  |  |
| **Stress Recognition** |  |  |  |
| When my workload becomes excessive, my performance is impaired |  |  |  |
| I am less effective at my clinical area when fatigued. |  |  |  |
| I am more likely to make errors in tense or hostile situations |  |  |  |
| Fatigue impairs my performance during emergency situations (e.g., emergency resuscitation, seizure) |  |  |  |
